# Kinetic and Regulatory Properties of *Yarrowia lipolytica* Aconitate Hydratase as a Model-Indicator of Cell Redox State under pH Stress

**DOI:** 10.3390/ijms24087670

**Published:** 2023-04-21

**Authors:** Tatyana I. Rakhmanova, Varvara Yu. Sekova, Natalya N. Gessler, Elena P. Isakova, Yulia I. Deryabina, Tatyana N. Popova, Yevgeniya I. Shurubor, Boris F. Krasnikov

**Affiliations:** 1 Department of Medical Biochemistry and Microbiology, Biology and Soil Science Faculty, Voronezh State University, Universitetskaya pl., 1, 394000 Voronezh, Russia; rtyana@mail.ru (T.I.R.); biomed-popova@yandex.ru (T.N.P.); 2 A.N. Bach Institute of Biochemistry, Research Center of Biotechnology of the Russian Academy of Sciences, Leninsky Ave. 33/2, 119071 Moscow, Russia; beauveria606@gmail.com (V.Y.S.); gessler51@mail.ru (N.N.G.); elen_iss@mail.ru (E.P.I.); 3 Centre for Strategic Planning of FMBA of the Russian Federation, Pogodinskaya St., Bld.10, 119121 Moscow, Russia; eshurubor@cspmz.ru

**Keywords:** yeast, aconitate hydratase, ambient pH, pH stress, kinetic parameters

## Abstract

This paper presents an analysis of the regulation activity of the partially purified preparations of cellular aconitate hydratase (AH) on the yeast *Yarrowia lipolytica* cultivated at extreme pH. As a result of purification, enzyme preparations were obtained from cells grown on media at pH 4.0, 5.5, and 9.0, purified by 48-, 46-, and 51-fold and having a specific activity of 0.43, 0.55 and 0.36 E/mg protein, respectively. The kinetic parameters of preparations from cells cultured at extreme pH demonstrated: (1) an increase in the affinity for citrate and isocitrate; and (2) a shift in the pH optima to the acidic and alkaline side in accordance with the modulation of the medium pH. The regulatory properties of the enzyme from cells subjected to alkaline stress showed increased sensitivity to Fe^2+^ ions and high peroxide resistance. Reduced glutathione (GSH) stimulated AH, while oxidized glutathione (GSSG) inhibited AH. A more pronounced effect of both GSH and GSSG was noted for the enzyme obtained from cells grown at pH 5.5. The data obtained provide new approaches to the use of *Y. lipolytica* as a model of eukaryotic cells demonstrating the development of a stress-induced pathology and to conducting a detailed analysis of enzymatic activity for its correction.

## 1. Introduction

Mammalian aconitate hydratases (aconitases, AH), represented by mitochondrial and cytosolic isoenzymes, are unique proteins containing iron–sulfur clusters where the metal center participates in the catalysis of the non-redox reaction [1]. In the Kuban iron–sulfur cluster (4Fe-4S) of aconitases, only three of the four iron ions have cysteine thiolate ligands, and the fourth one (Feα) is located in the pocket of the active center and bound with either the oxygen of water or substrates to be dehydrated. The catalyzed reaction is a reversible isomerization of citrate into isocitrate with an intermediate metabolite of cis-aconitate.

There are cytoplasmic and mitochondrial AHs differing according to their physical, chemical, and structural properties. Both AH isoenzymes have iron–sulfur clusters (4Fe-4S) bound to cysteine residues of Cys437, Cys503, and Cys506 [2]. The cytosolic form of AH (cAco, ACO1) is an enzyme, which turns into the iron regulator protein 1 (iron modulatory protein, IRP1) when it loses the iron–sulfur cluster [3]. When the iron–sulfur cluster formation is dysregulated, ACO1 is converted into IRP1, which decreases the synthesis of 2-oxglutarate, disrupting the regulation of TCA and iron metabolism. IRP1 is a powerful bifunctional enzyme also acting as ACO1 [4]. As soon as the intracellular concentration of iron increases above normal, ACO1 binds to the iron–sulfur cluster (4Fe-4S) and functions as an aconitase. At the same time, when the level of intracellular iron becomes lower than the acceptable level, IRP1 serves as a protein regulating the level of iron after it leaves the iron–sulfur cluster. [5,6]. In addition to IRP1, the maintenance of cellular and systemic iron homeostasis in the cell is performed by another iron modulatory protein, IRP2, which is also sensitized under the conditions of iron deficiency [7]. IRP1 is extremely sensitive to small changes in the concentrations of oxygen, nitrogen oxides, and hydroxides. Thus, ACO1 can turn into IRP1 when the oxygen level increases, and its RNA-binding activity can rise significantly. However, IRP2 is sensitive to the iron concentration and could be activated at its deficiency in the cell [3]. The mitochondrial isoform of AH (mAco, ACO2) catalyzes the second reaction in the Krebs cycle, also playing an important role in lipid metabolism. Citric acid is exported through the mitochondrial membrane to the cytosol and then converted into oxaloacetate and bicarbon acetyl-CoA, which is a precursor for the synthesis of fatty acids. Thus, ACO2 is an important enzyme which links the TCA cycle and lipid metabolism [8]. It is assumed that AH isoenzymes, catalyzing the same reaction, perform multidirectional physiological functions in the cellular metabolism, reflecting the influence of the enzyme on oxidative and biosynthetic processes. Thus, the reaction catalyzed by mitochondrial AH is one of the initial stages of TCA, while the cytoplasmic AH function is mainly aimed at regulating the citrate accumulation and degradation in the lipogenesis [9].

Having a cluster (4Fe-4S) in its composition, AH can be exposed to the effect of some cell radicals. In recent decades, numerous experimental data have shown that AH are the main targets for reactive oxygen (ROS) and nitrogen (RNS) species, namely superoxide radicals (O_2_^•–^), hydrogen peroxide (H_2_O_2_), nitric oxide (^•^NO), and peroxynitrite (ONOO^–^). These radicals are capable of oxidizing the enzyme iron–sulfur cluster, which leads to the release of iron and, as a consequence, the loss of the catalytic activity of AH [5]. While ^•^NO reacts moderately with AH, its reaction with O_2_^•–^ causes the generation of ONOO^–^, which induces the destruction of the cluster (4Fe-4S). In the case of ACO1, it was noted that H_2_O_2_ and ^•^NO facilitate the activation of iron-sensitive proteins in the cells in vitro. AHs are also the main targets for cellular radicals in the models in vivo, demonstrating such posttranslational oxidative modifications as S-nitrosylation and carbonylation of proteins [1,5].

The O_2_^•–^-induced suppression of AH activity is well known. This makes it possible to consider the enzyme as a sensitive and crucial target for ROS action during oxidative stress [10,11,12]. Depending on the intensity and duration of the oxidative stress, AH may be reversibly inhibited due to the oxidation of cysteine residues, and then it can be irreversibly inactivated due to the cluster disassembly (4Fe-4S), carbonylation, and ATP-dependent degradation. Recently, interest in the study of the regulation and catalytic features of AH has increased due to abundant information on the relationship between the enzyme functional activity and some pathological cellular processes. Thus, the disturbance of iron homeostasis is reported to be an essential metabolic sign of cancer cells [13]. Some studies showed the ectopic overexpression of *ACO1* and *IRP1* in some types of cancer [14,15,16]. In the development of prostate cancer, AH plays a key role in the context of tumor physiology. In normal prostate tissue, high level of zinc can inhibit the AH activity causing the citrate accumulation. To the contrary, in the prostate cancer cells, the enzyme activity is restored, which leads to increased citrate oxidation followed by a decrease in fatty acid synthesis [17]. Some changes in the mitochondrial AH activity were noted in colorectal cancer [8]. Moreover, the pathogenic variants of the mAco *ACO2* gene are responsible for numerous pathologies associated with optic nerve degeneration, ranging from neuropathy to complex neurodegenerative syndromes. [18].

Yeasts are often used as convenient models of unicellular eukaryotes and have gained wide popularity in experiments simulating a stressful cellular state. The extremophilic yeast *Yarrowia lipolytica* is an excellent tool for analyzing the adaptation of ROS-sensitive enzymatic systems at hyper-oxidation due to the oxidative stress. This yeast strain is capable of adapting to various external stressors and prospers in environments with extremely high (up to pH 9.5) and low (pH 3.0) pH [19,20], as well as under the conditions of low water activity (high salinity, dry and hydrophobic substrates) [21]. It should be noted that alkalotolerance (resistance to the alkaline conditions) is not a typical property for yeast, since most of them grow at an optimum pH of 5.5–6.5 and are rarely able to withstand alkaline stress up to a pH of 8.0 [22]. The information on the AH regulation in yeast is limited. It is known that in the yeast *Saccharomyces cerevisiae*, knockout of the *ACO1* (Δaco1) gene encoding the cytosolic isoform of the enzyme is known to lead to the disturbance of the mitochondrial DNA [23]. This gave reason to assume that AH can directly interact with DNA, maintaining its stability. [24]. In the fission yeast of *Schizosaccharomyces pombe*, it was shown that ACO2 binds directly to the mRNAs of the iron carriers (to the mRNAs of iron uptake transporters), thus regulating its homeostasis at the genetic level [25]. The results of our studies using the *Endomyces magnusii* yeast showed that the total AH activity increased significantly in the late stationary growth phase and was not inhibited during the long-term cultivation for seven days. [26]. Some recent studies confirmed the sensor role of AH under the conditions of pro-oxidant effects [27,28,29,30].

In the present study, we analyzed the regulation of the cell AH activity in the *Y. lipolytica* yeast at extreme pH values.

## 2. Results

### 2.1. Growth, ROS Generation, and Respiratory Activity of the Y. lipolytica Yeast under Different Ambient pH

**Figure 1:**
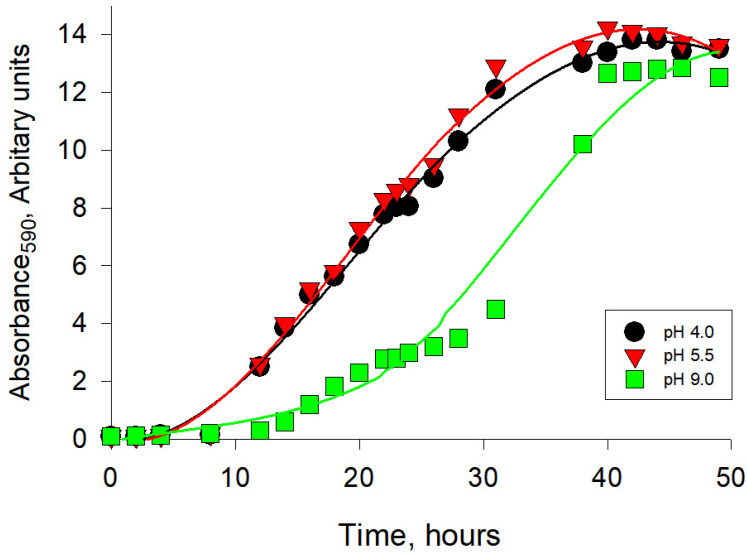
The effect of ambient pH on the growth of *Y. lipolytica* W29 in glycerol-containing (1%) medium. Absorbance was assessed in cell suspensions at the wavelength of 590 nm (A590).

Previously, we have shown that the optimum pH for the growth of the *Y. lipolytica* yeast is within usual yeast optimal pH interval of 4.5–6.0 [31]. The growth in the pH range from 3.5 to 4.5 showed a smooth rise on the curve, a slight jump at pH of 5.0, and a gradual decrease in growth features at pH of 10.5–11.0. The shift in growing conditions towards alkaline pH values (pH ≥ 7.5) in relation to the optimal pH led to a more than 4-fold decrease in the value of the calculated biomass doubling rate (μ). However, a long stage of logarithmic growth of the culture made it possible to increase the volume of cell mass, comparable with the accumulation of biomass under optimal conditions for the strain. This gives grounds to classify the *Y. lipolytica* W29 strain studied by us as a moderate alkalotolerant [32]. At the same time, the range typical for most yeasts, 5.5–6.0, can be considered optimal for the growth of *Y. lipolytica*. The drop in biomass accumulation was 20% and 60% when the yeast was cultivated at pH 4.0 and 9.0, respectively. The plot of absorbance of the *Y. lipolytica* cell suspension versus culture pH presented in Figure 1 shows the overall pattern of long-term yeast growth to the stationary phase and demonstrates that pH variations significantly affect the length of the lag-period and the exponential phase of yeast growth. Thus, at pH 4.0 and 5.5, the duration of the lag period was less than 8 h, while at pH 9.0 it was about 13 h. In addition, we observed a slowdown in the culture growth rate at pH 9.0, which manifested itself in the appearance of an extended logarithmic phase (Figure 1). The onset of the stationary phase of growth at alkaline pH occurred 10 h later than at pH 4.0 and 5.5. An analysis of the growth characteristics testified to the development of a stress effect on cells at extreme pH of the medium, which was especially pronounced at alkaline values.

**Figure 2:**
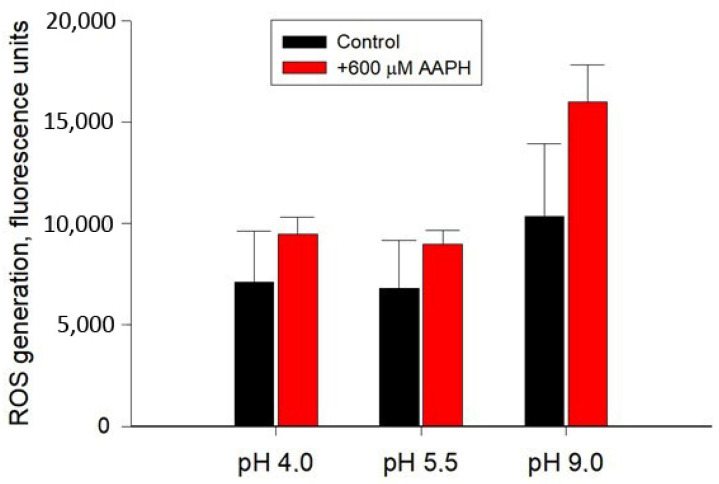
Fluorescence intensity (units), reflecting total ROS generation, 60 min after cell suspension staining with *Y. lipolytica* W29 H_2_DCF-DA at various pH values. Cells exposed to pro-oxidant 600 μM 2,2′-azobis(2-methylpropionamidine) dihydrochloride (AAPH) were used as a positive control. The incubation medium for the experiments contained 50 mM KPi, pH 5.5; and 1% glucose. Values are mean ± standard deviation from 5–6 independent experiments. No statistically significant differences compared to the corresponding control.

As is known, any stress effect on a cell can result in the development of oxidative stress, a critical imbalance between the formation and inactivation of reactive oxygen species (ROS). Therefore, we further investigated the overall level of production of the superoxide anion radical as the most reactive oxygen compound in *Y. lipolytica* cells grown at different pH values. Cells exposed to 600 μM 2,2′-azobis(2-methylpropionamidine) dihydrochloride (AAPH), a potent pro-oxidant, were used as a positive control. The results of the study are shown in Figure 2. As can be seen, the highest level of ROS generation, 1.5 times higher than this parameter in the control (although not statistically significant), was observed in cells grown under alkaline conditions, while in cells cultivated at acidic pH values, this parameter remained virtually unchanged. The positive control in the presence of 600 μM AAPH was characterized by a similar level of ROS production (Figure 2), which indirectly indicated a high degree of constitutive production of oxygen radicals by cells.

The level of free radical generation is directly related to the aerobic metabolic activity of cells, the main parameter of which is cellular respiration. The total oxygen consumption by cells of a number of eukaryotes (yeast, fungi, and plants) is contributed to by two main blocks of enzyme complexes responsible for electron transfer in the inner mitochondrial membrane: the main (cytochrome pathway) and the alternative (cyanide-resistant pathway) oxidases [33]. The contribution of alternative oxidase to the overall rate of cellular respiration can be regarded as one of the markers of oxidative stress, since it is known that ROS cause peroxidation of cardiolipin, which is necessary for the functioning of cytochrome oxidase. A similar state is achieved when cells are treated with KCN. Thus, the cell is forced to switch to an alternative oxidation pathway [34].

**Figure 3:**
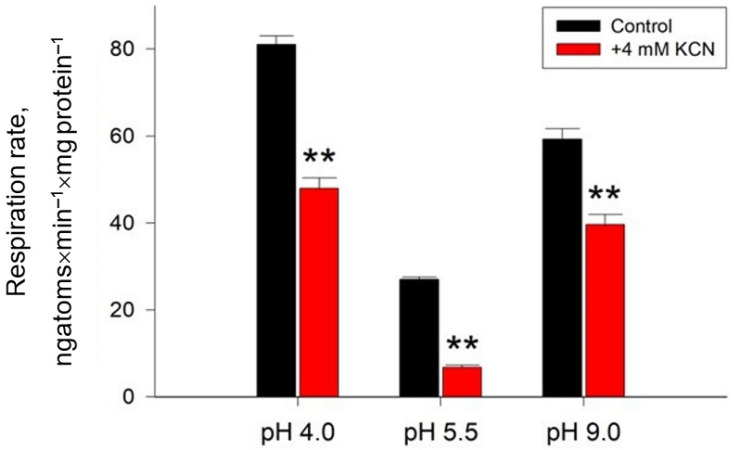
Respiratory activity and alternative oxidase induction of the *Y. lipolytica* W29 yeast at various pH values. Values are mean ± standard deviation from 5–6 independent experiments. **—Statistically significant difference compared to the corresponding control, *p* < 0.005.

Data on the rate of cell respiration and the contribution of alternative oxidase to it are shown in Figure 3.

The most intensive respiration, exceeding the values at normal pH by 3 and 2 times, was observed at pH 4.0 and 9.0, respectively (Figure 3). At the same time, the activity of the alternative oxidase increased by more than 2 times under any type of stress, reaching almost 70% of the total cellular respiration in the case of alkaline conditions (Figure 3). Together with a reduced value of µ under these conditions, this could indicate pronounced oxidative stress and reduced metabolic activity of the culture. It should be noted that an increase in the rate of respiration along with an increase in the contribution of alternative oxidase is a well-known phenomenon for the yeast *Y. lipolytica*, which was first shown in 1999 [35]. Switching respiration to an alternative electron transport pathway is less efficient for ATP synthesis, but not for oxygen consumption. Considering that seven alternative oxidase genes encoding at least nine protein variants are known for *Y. lipolytica* [36], the induction of alternative oxidase under stress conditions or upon inhibition of the main electron transport pathway can occur quickly enough not only not to reduce the rate of oxygen consumption, but also in some cases to increase it [37].

Thus, a preliminary analysis of the redox and energy status of *Y. lipolytica* cells under extreme pH conditions showed the development of a pronounced adaptive response of yeast cells to stress.

### 2.2. Isolation and Purification of Aconitate Hydratase from the Y. lipolytica Yeast Grown at Different pH

To analyze the features of the catalytic action of the enzyme upon the adaptation of the *Y. lipolytica* yeast to an extreme pH compared to those in the culture grown under normal conditions, we designed a method for purifying the enzyme from the yeast cells. Upon developing the method, it turned out that passing it through sephadex G-25 significantly decreased the activity of AH, which was obviously associated with the damage caused to the enzyme structure during the gel filtration. Thus, some studies report the alterations in the initial structure of the (Fe-S) cluster of the AH due to the dissociation of the iron atom involved in the substrate binding from the active center of the enzyme upon either gel filtration or dialysis of AH preparations from animal tissues [38]. It turned out that the same pattern is typical for the enzyme from the *Y. lipolytica* yeast. The application of Fe^2+^ at a concentration of 10 µmol/L to the elution medium prevented the decrease in the enzyme activity during the gel filtration chromatography on G-25. Further purification of the enzyme was performed using ion exchange chromatography on a column with DEAE cellulose (1.2 × 13 cm). After the protein sorption, the enzyme was desorbed on the column using a stepwise KCl gradient in the same elution medium. The column was washed with 100 mM KCl, which permitted us to remove the associated proteins at this stage. The enzyme was desorbed using a 200 mM KCl gradient. The designed conditions for the AH purification resulted in obtaining the enzyme samples from the yeast cells cultured at pH of 4.0, 5.5, and 9.0, which were purified 48-, 46-, and 51-fold, respectively. The specific activity was 0.43, 0.55, and 0.36 U/mg of protein, respectively (Table 1). 

The AH samples purified in this way were used to study the kinetic parameters of the catalytic action of the enzyme. The parameters of the enzyme yield and the degree of its purification indicated that a partially purified sample of high instability was obtained. Storage of the enzyme sample at a temperature of 2 °C decreased the enzyme activity by 85% for a day. The addition of glycerol at a concentration of 25% of the total volume stabilized no enzymatic activity. 

### 2.3. Study of the Kinetics of the Reaction Catalyzed by AH from Y. lipolytica Cells Grown on Media with Different pH Values

**Figure 4:**
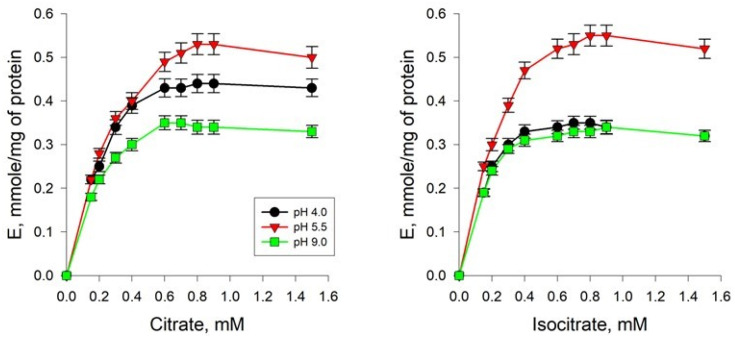
Dependence of the rate of the enzymatic reaction on the concentration of the substrates of citrate and isocitrate, determined for AH isolated from the *Y. lipolytica* cells grown at pH of 4.0, 5.5, and 9.0. Values are mean ± standard deviation from 5–6 independent experiments.

The AH preparations obtained after the final stage of purification from *Y. lipolytica* cells of the experimental variants were used to study the dependence of the rate of the enzymatic reaction on the concentration of the substrates of the forward and reverse reactions: citrate and isocitrate, respectively. According to the results of the study, it was revealed that the kinetics of the reaction catalyzed by AH is described by the Michaelis–Menten equation (Figure 4).

**Figure 5:**
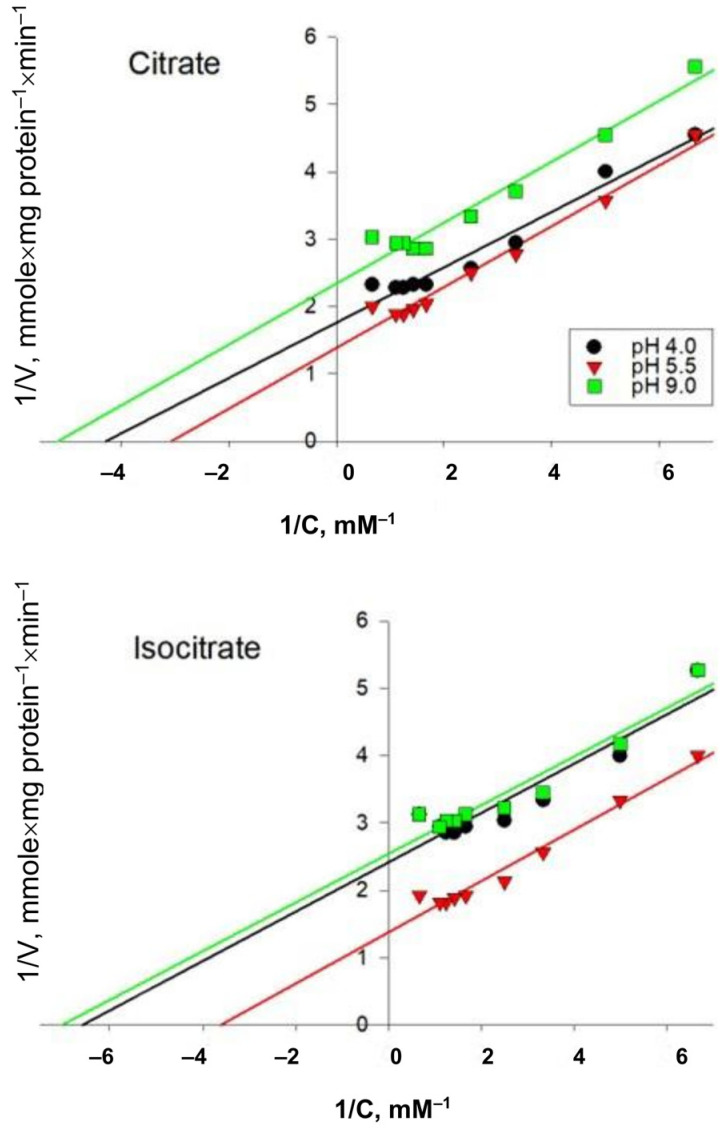
Dependence of the enzymatic reaction rate on the concentration of citrate and isocitrate in Lineweaver–Burk coordinates for AH isolated from *Y. lipolytica* cells grown at pH 4.0, 5.5, and 9.0.

The Michaelis–Menten constants for citrate and isocitrate for the enzyme from the *Y. lipolytica* cells cultured at different pH were calculated using double inverse Linewiver-Burke coordinates (Figure 5). As can be seen from Figure 5, for different pH values, the Lineweaver–Burk trend lines are parallel to each other and to the control conditions, but the points of intersection with the y-axis are above zero.

When evaluating the maximum reaction rate, we found that it decreased under extreme pH conditions—by 20% and 40% on citrate as a substrate and by 40% and 50% on isocitrate at acidic and alkaline pH, respectively (Table 2). Furthermore, it was found that the concentration of citrate at which the rate of the reaction catalyzed by AH is equal to half the maximum was 0.32 mM under conditions of normal pH values of the *Y. lipolytica* cultivation medium. However, when yeast cells were cultivated on a medium with extremely low and extremely high pH values, an increase in the affinity of the enzyme for this substrate was observed, as evidenced by a decrease in the Michaelis–Menten constant by 1.39 and 1.68 times, respectively (Table 2). Regarding the affinity of the enzyme for isocitrate, we found that the K_m_ for this substrate decreased by 1.8 times when growing *Y. lipolytica* under conditions of extremely low pH values and by 1.9 times under conditions of extremely high pH values relative to the same parameter for yeast cells grown under pH 5.5 conditions (0.27 mM) (Table 2). Thus, adaptation of *Y. lipolytica* to extreme pH values of the medium resulted in an increase in the affinity of the enzyme for the substrate both in the forward and reverse reactions.

### 2.4. Study of the Influence of the Concentration of Hydrogen Ions on the Rate of the Enzymatic Reaction Catalyzed by AH Isolated from Yeast Cells under Different Cultivation Conditions

**Figure 6:**
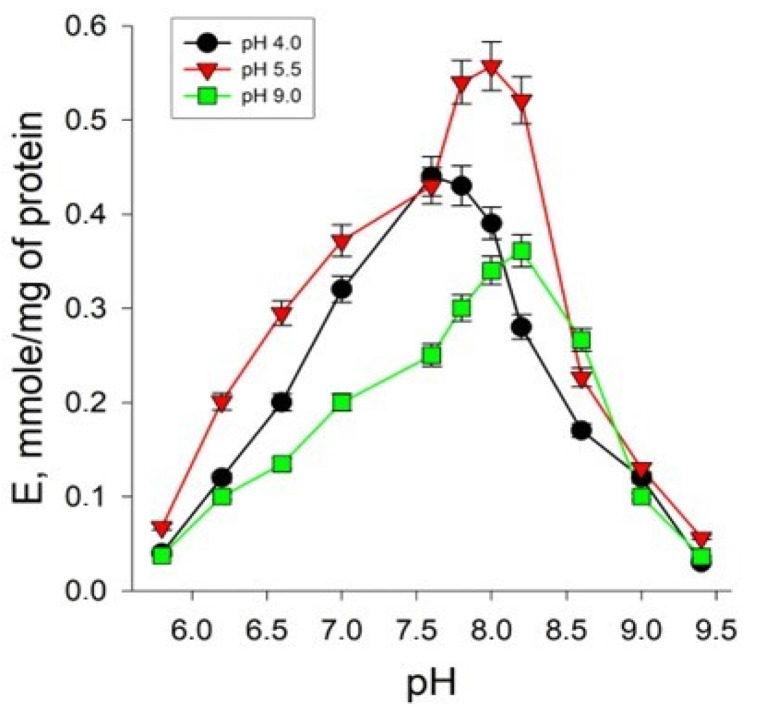
Hydrogen ion concentration dependence of the enzymatic reaction rate catalyzed by AH isolated from *Y. lipolytica* cells grown at pH 4.0; pH 5.5; and pH 9.0. Values are mean ± standard deviation from 5–6 independent experiments.

The study of the dependence of the rate of the AH reaction on the concentration of hydrogen ions showed that both under normal and under extreme conditions of cultivation, the enzyme was active in the pH range from 6.0 to 9.0. The pH optimum for AH from *Y. lipolytica* cells grown under normal conditions was 8.0. The cultivation of yeast cells in extremely acidic conditions of the nutrient medium was accompanied by a decrease in the optimum pH for AH to 7.6. However, when growing yeast under conditions of extremely high concentrations of hydrogen ions in the medium, a statistically insignificant increase in the optimum pH of AH to 8.2 was observed compared to normal cultivation conditions (Figure 6).

### 2.5. Regulatory Properties of AH from Y. lipolytica Cells Grown on Media with Different pH Values

According to the literature data, the following ways of regulating AH activity are known: (1) activation/inactivation due to the assembly–disassembly of the iron–sulfur cluster or the replacement of iron in it by other metals; (2) inactivation due to modification of cysteine and tyrosine residues; and (3) competitive inhibition by di- and tri-carboxylic acids. Therefore, at the next stage of the work, we evaluated the effect of Fe^2+^ ions on the activity of the enzyme from yeast cells. In addition, given that both AH isoenzymes have a [4Fe-4S] cluster associated with the cysteine residues Cys437, Cys503, and Cys506 in the active site [2], it seemed interesting to study the effect of the oxidized and reduced forms of glutathione, as well as the peroxide radical, which can change the state of Cys residues to the activity of the enzyme. The activity of AH in the culture grown at pH 4.0 showed a trend almost identical without statistically significant differences to that at pH 5.5. Due to this fact, the data on AH in the yeast at pH 4.0 are not included so as not to clutter up the picture.

#### 2.5.1. Effect of Fe^2+^ Ions

**Figure 7:**
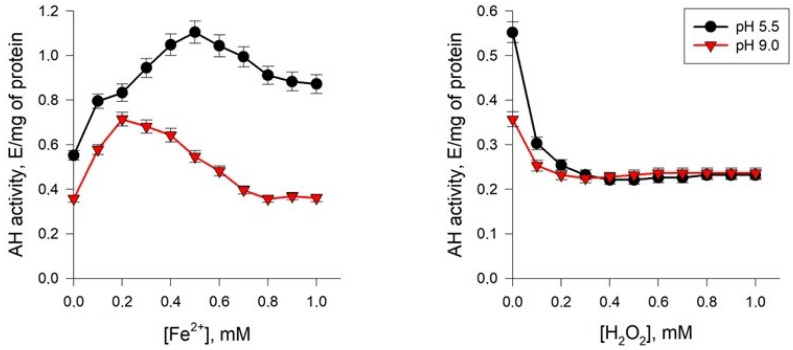
Effect of iron ions (Fe^2+^) and H_2_O_2_ on the activity of AH from *Y. lipolytica* under cultivation conditions at pH 5.5 (Black circles) and 9.0 (Red triangles).

Studies of the effect of Fe^2+^ ions on the activity of AH from *Y. lipolytica* showed that these ions increased the activity of the enzyme both under normal pH conditions and at extremely alkaline values (Figure 7). However, in the latter case, AH was more sensitive to the activating effect of Fe^2+^ than under normal conditions. Thus, a 2-fold increase in the rate of the AH reaction at a medium pH of 9.0 was observed at Fe^2+^ concentrations up to 0.2 mM, and under normal conditions—0.5 mM. A further increase in concentration led to a slight decrease in activity.

#### 2.5.2. Effect of H_2_O_2_

AHs are known to be the main targets of the reactive oxygen (ROS) and nitrogen (RNS) species, which can oxidize the iron–sulfur cluster of the enzyme. It leads to the release of iron and, as a consequence, to the loss of the AH catalytic activity [5]. Therefore, we assayed the dynamics of the AH activity depending on the action of peroxide radicals at different concentrations. We have shown that H_2_O_2_ reduced the AH activity (Figure 7). The enzyme from the yeast cells cultured under normal conditions proved more sensitive to the inhibitory effect of peroxide (Figure 7). Thus, H_2_O_2_ at a concentration of 0.2 mM decreased the enzyme activity by more than 50% in the culture grown at pH of 5.5 and decreased it by 35% in the yeast cultivated at pH 9.0 (Figure 7). Upon a further increase in the metabolite-regulator level up to 1 mM, the inhibitory effect persisted.

#### 2.5.3. Effects of Glutathione

**Figure 8:**
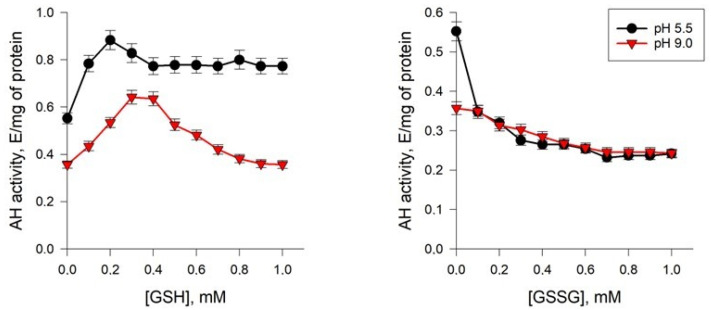
Effect of GSH and GSSG on the activity of *Y. lipolytica* AH under cultivation conditions at pH 5.5 (Black circles) and 9.0 (Red triangles).

Some studies have shown that upon adaptation of *Y. lipolytica* to the extreme pH, some changes in the level of both oxidized and reduced glutathione in the yeast cells were observed. In this regard, we evaluated the effects of the glutathione antioxidant system on the AH activity. The study revealed significant changes in the regulation of AH activity upon the action of GSH and GSSG (Figure 8).

The activity of the enzyme from the yeast cells grown at pH 9.0 and 5.5 increased when the level of the reduced glutathione rose up to 0.3 and 0.2 mM, respectively. At higher concentrations, the degree of the inducing effect decreased (Figure 8). So, a more significant increase in the AH activity was detected for the enzyme from the cells cultured at pH of 5.5. The oxidized glutathione inhibited AH activity from the cells of all of the groups tested, affecting more strongly the enzyme isolated from the culture grown at pH 5.5. Thus, at a concentration of the oxidized glutathione of 0.2 mM, the enzyme activity decreased by more than 40% when the population was cultivated at pH 5.5 and by 12% when it was cultivated at pH 9.0 (Figure 8). A further increase in the metabolite level up to 1 mM facilitated the inhibitory effect for both cultures.

## 3. Discussion

Non-enzymatic free radical oxidation is an important factor in the development of oxidative stress in aerobic cells. Free radicals are involved in redox reactions leading to oxidative modifications in biomolecules, among which proteins and lipids occupy an important place [39]. It is known that the main reasons for the activation of free radical oxidation under oxidative stress, in addition to an increase in the production of ROS, are the release of iron ions from extra- and intracellular depots [38]. An increase in the intracellular concentration of iron ions can occur as a result of the breakdown of Fe-containing proteins, in particular, AH [10,40]. In our work, we attempted to analyze the regulatory properties of partially purified AH preparations from *Y. lipolytica* yeast cells cultivated under conditions of extremely high and low pH values. We found a significant decrease in enzyme activity under extreme pH conditions, both in forward and reverse reactions (Table 2, Figure 5), which could indicate the partial inactivation of enzyme functioning under hyperoxidation conditions. An assessment of the kinetic parameters of the catalytic action of AH showed that during the cultivation of yeast cells on a medium with extremely low and extremely high pH values, an increase in the affinity of the enzyme for citrate was observed, as evidenced by a decrease in the Michaelis–Menten constant by 1.3 and 1.68 times, respectively (Table 2, Figure 5). For comparison, the K_m_ of AH from the porcine heart for citrate is 7.3 µM, and the K_m_ of mitochondrial and cytoplasmic AH from white maple protoplasts for citrate is 120–130 µM [38]. The K_m_ for citrate of cytoplasmic AH from rat liver is 0.42 mM, and mitochondrial AH is 0.48 mM, which is comparable with our data. It has been shown that the K_m_ values for citrate and isocitrate for AH isolated from *Corynebacterium glutamicum* are 0.48 mM and 0.55 mM, respectively [41]. Regarding the affinity of the enzyme for isocitrate, we found that the K_m_ for this substrate decreased by 1.75 times when growing *Y. lipolytica* under conditions of extremely low pH values and by 1.84 times under conditions of extremely high pH values relative to the same parameter for yeast cells grown at pH 5.5 (0.32 mM) (Table 2, Figure 5). Thus, we have shown that the adaptation of *Y. lipolytica* to extreme pH values resulted in an increase in the affinity of the enzyme for the substrate both in the forward and reverse reactions. It should be emphasized that V_max_ and K_m_, shown in Figure 5 and presented in Table 2, change similarly to the situation that would be observed in the presence of an uncompetitive inhibitor. It is known that uncompetitive inhibitors stabilize the substrate enzyme and reduce the rate of catalysis [38,41]. Based on the fact that the nature of the curves does not change at different pH values of the medium, it can be assumed that changes in the concentration of hydrogen ions in the medium cause the substrate to remain bound longer due to a decrease in the k_cat_ of the enzyme, which reduces the release of the product.

The study of the pH dependence of the AH reaction rate showed the presence of a wide optimum in the pH range from 6.0 to 9.0. The pH optimum values for AH from many organisms available in the literature also lie within this range. Thus, the pH optimum for the functioning of AH from a pig heart is 7.8 [42]. Brouqusisse et al. showed that for the cytoplasmic and mitochondrial enzymes isolated from the culture of white maple cells, the pH values are 7.1 and 7.4, respectively [43]. Brouquisse et al. set the pH optima for AH from cell suspensions of plane tree (*Acer pseudoplatanus*), equal to 7.1–7.4 [44]. An interesting observation was the shift in the pH optimum of AH depending on the cultivation conditions—for AH from *Y. lipolytica* cells grown under normal conditions, it was 8.0, for those grown under extremely acidic conditions it was 7.6, and for those grown under conditions of extremely high pH values it was 8, 2 (Figure 6). Differences in the pH optima for the enzyme in experimental variants, along with a change in the degree of affinity to substrates, can probably determine the possibility of shifting the equilibrium of the AH reaction, affecting the rates of the “forward” and “reverse” reactions, which may be important when adapting to extreme cultivation conditions. This explanation is partly supported by the fact that under conditions of extreme pH, yeast cells maintain a high energy level and increased metabolic activity (Figure 3), which may indicate the efficient functioning of AH in both cytoplasmic and mitochondrial localization. It is also possible that the intracellular pH of yeast may change within a certain range with fluctuations in the pH of the external environment.

As was mentioned above, the following three ways of regulating AH activity are known: (1) activation/inactivation due to the assembly–disassembly of the iron–sulfur cluster or the replacement of iron in it by other metals; (2) inactivation due to modification of cysteine and tyrosine residues; and (3) competitive inhibition by di- and tri-carboxylic acids. AH expression can be regulated at the posttranscriptional level [43]. At the same time, it is noted that an important feature in the regulation of AH activity is the presence of Fe^2+^ ions, and ions of other metals cannot replace them. In the absence of Fe^2+^, only 70% of the level of maximum AH activity is achieved [44]. Studies of the effect of Fe^2+^ ions on the activity of AH from *Y. lipolytica* showed that Fe^2+^ ions increased the activity of the enzyme both under normal pH conditions and at extremely alkaline values (Figure 7), and in the latter case, an increase in the rate of the AH reaction by 2 times at a medium pH, 9.0, was observed at Fe^2+^ concentrations more than 2 times lower than at normal pH. According to references, at a low iron concentration in the cell cytosol, there is apo-aconitase carrying the [3Fe-4S] cluster. Upon the iron concentration increase in the cytoplasm, apo-aconitase acquires an additional iron atom and evolves into the holo-aconitase, already being in an active form. Both the mitochondrial and cytosolic forms of AH perform a dual function mediated by the Fe-S cluster. [45]. The differences in the degree of the sensitivity of the enzyme isolated from the cells grown at different pH may result from the changes in AH microenvironment, which accompanies the oxidative stress at pH 9.0. Moreover, the information for the enzyme from the porcine myocardium, where the isomorphic incorporation of Fe^2+^ into the 3Fe-4S clusters of the inactive part of the molecule is associated with a slow conformational shift, can confirm the obtained data. Some papers discuss the physiological role of the effect of “acceptance” of free Fe^2+^ ions by AH, which involves maintaining a constant level of the ions in the subcellular compartments [46]. It can be assumed that there is a possible relationship between the revealed difference in the AH activation and changes in the local concentration of iron (II) ions in the cells upon the cultivation at pH 9.0. Our data indicate changes in metal metabolism under pH stress conditions. This phenomenon has been described for yeast cells under acid stress. Analysis of gene expression using microarrays revealed that acid stress leads to changes in the expression of genes regulated by transcription factors Aft1p and Aft2p, namely iron reductases FRE1-3, FRE5, iron permease FTR1, siderophore transporters ARN1 and 2, ferroxidase (FET3) and proteins associated with the uptake of FIT1–FIT3 siderophores [47,48,49,50]. Similar phenomena were also observed under conditions of iron deficiency. The two most likely explanations for this are that, under pH stress conditions, (1) cells cannot import iron and (2) cells need more iron. It is known that iron metabolism is determined to a greater extent not by the external concentration of iron, but by the activity of iron–sulfur clusters, which in yeast are localized mainly in the mitochondria. The results of some studies show that the level of the proteins containing iron–sulfur clusters encoded by the *LEU1* and *ACO1* genes significantly decrease under acidic conditions [51]. On the other hand, the negative effect on the cells of an alkaline pH includes reducing the solubility of some crucial trace elements, namely iron and copper. The regulation of iron homeostasis in fungi has been mainly studied using the *S. cerevisiae* model. The transcriptional activator of Aft (Aft1 and Aft2) expresses the genes involved in iron uptake if its availability is limited [51]). It is mediated by the Fe-S clusters synthesized in the mitochondria, which bind the glutaredoxins Grx3 and Grx4 and allow them to interact with Aft1 to remove it from its promoter targets [51]. Such Fe-S clusters also play a role in the adaptation to high iron concentrations. The high sensitivity of the enzyme isolated from the cells under alkaline stress to iron ions, as shown in our study, may be related to the microelement deficiency and interaction with the transcription activators. On the contrary, the introduction of the additional copies of the *FET4* and *CTR1* genes increased the population survival. Moreover, an increase in the concentration of iron and copper ions increased the resistance of *S. cerevisiae* to high pH [52]. This assumption is confirmed by the fact that the deletions of some genes associated with iron and copper metabolism (*CCC2, AFT1, FET3, LYS7,* and *CTR1*) decreased the cell survival under the alkaline conditions [53]. Thus, an increase in the concentration of iron and copper ions is shown to facilitate the resistance of *S. cerevisiae* to alkaline pH [53].

As is known, both Fe^2+^ and H_2_O_2_ ions are components of the Fenton and Haber–Weiss reactions, during which the most reactive form of ROS, OH^•^, is formed, which leads to the initiation of free radical oxidation processes and causes the disturbance of oxidative homeostasis under stress conditions [54]. We showed that H_2_O_2_ decreased AH activity (Figure 7). The enzyme from yeast cells cultivated under normal conditions turned out to be more sensitive to the inhibitory effects of peroxide (Figure 7). Taking into account the increased production of ROS under alkaline pH conditions (Figure 2), which we showed for *Y. lipolytica* yeast cells, it can be assumed that an increased free radical background under pH stress conditions induces an increase in the resistance of the enzyme to peroxide exposure due to the combined protective action of antioxidant cell systems. In particular, we have previously shown that, under alkaline pH conditions, the activity of superoxide dismutase increased by almost six times [31]. A decrease in the level of superoxide anion generation could probably reduce the degree of sensitivity of AH to free radical action. There is evidence that the inactivation of AH from the liver of rats under normal conditions and during the development of toxic hepatitis, which was accompanied by oxidative stress and increased ROS formation, under the action of hydrogen peroxide is reversible and proceeds according to a mixed type of inhibition [55]. The regulation of the rate of citrate conversion by hydrogen peroxide may be important in preventing the formation of the hydroxyl radical generated during the Fenton reaction.

The activity of AH from yeast cells increased with an increase in the concentration of reduced glutathione and decreased when exposed to its oxidized form (Figure 8), with a more pronounced effect on the enzyme under normal yeast cultivation conditions, while at pH 9.0, the activity decreased by only 12%. The regulatory effect of glutathione in the oxidized and reduced forms on the activity of AH can be explained by the influence of its functional SH-groups on the redox state of Fe^2+^ in the active center of the enzyme. There are some results in the literature indicating that the modification of cysteine residues in or near the active site with N-ethylmaleimide inhibits AH [56]. It is assumed that the cysteine residues are involved in the regulation of the enzyme activity depending on the cell redox status of thiols. Thus, the induction of the enzyme by the reduced glutathione can be explained by the reduction in the oxidized cysteine residues. However, upon increasing the concentration of glutathione, glutathionylation of cysteine residues with the formation of mixed disulfides dominates, leading to a decrease in the activity [57]. It is also possible that significant activation of the enzyme under the action of glutathione can stimulate the functioning of the enzyme, which is associated with the supply of isocitrate for NADP-isocitrate dehydrogenase (NADP^+^-dependent IDH). NADP^+^-dependent IDH belongs to the class of IDs that catalyze the oxidative decarboxylation of D,L-threo-Ds-isocitrate to 2-oxoglutarate. The function of NADP^+^-dependent IDH is predominantly involved in the biosynthetic processes of the cell [58]. The oxidation of isocitrate to 2-oxoglutarate catalyzed by NADP^+^-dependent IDH is closely related to the enzymatic transformations of GSSG and GSH (2GSH=GSSG+2H^+^) catalyzed by glutathione reductase (GLR). NADP^+^-dependent IDH was isolated from the *Y. lipolytica* CLIB122 (YlIDP) producer and is an enzyme with absolute specificity for NADP^+^, with a K_m_ for isocitrate of about 60 μM [59]. Thus, AH can be involved in a complex network of interactions both with the glutathione system and with enzymes that supply NADPH, which are necessary for its effective functioning, in particular, with the first enzyme of the pentose phosphate pathway, NADP-dependent glucose-6-phosphate dehydrogenase (G6PDH) [60], as well as with the activity of some enzymes providing alternative NADPH production, for example NADP-dependent IDH [59,61]. The proposed explanation is supported by the fact that at high concentrations of glutathione, AH activity decreased. Our previous studies have shown that the intracellular concentration of oxidized glutathione at alkaline pH was comparable to that obtained under normal conditions (4.15 μM/mg versus 3.62 μM/mg) [31]. This fact also confirms our assumption that, under conditions of alkaline stress, the functioning of the glutathione system is rearranged, which leads to a decrease in the sensitivity of AH to the effects of the oxidized form of the peptide.

## 4. Conclusions

Thus, as a result of our studies, we determined the kinetic and regulatory parameters of the action of yeast AH, which ensures the functioning of a number of catabolic and anabolic reactions associated with the accumulation and utilization of citrate under pH stress conditions. This made it possible to obtain new knowledge about the implementation of metabolic processes in *Y. lipolytica* cells during cultivation on a nutrient medium with extreme pH values. It should be noted that a violation of the pH balance can be the cause of a number of pathological conditions—both acidosis, which develops as a result of the increased production of acids, the reduced excretion (excretion) of acids and/or the increased excretion of bases [62,63], and alkalosis, accompanied by the accumulation of bases or the loss of acids [64]. The causes of metabolic acidosis are hypoxia, circulatory disorders, diabetes mellitus, and severe damage to the liver and kidneys. True metabolic alkalosis can develop as a result of damage to the adrenal cortex (with adenoma, carcinoma) and with a decrease in the function of the parathyroid glands. Such serious pathologies require detailed analysis at the cellular and molecular level. Our proposed approach to the use of the extremophilic yeast *Y. lipolytica* as a model of a eukaryotic cell, which makes it possible to simulate the development of stress conditions at the molecular level, making it possible to conduct a detailed analysis of enzymatic activity under pathological conditions in order to develop effective approaches to their correction.

## 5. Materials and Methods

### 5.1. Yeast Strains and Growth Conditions

The wild-type yeast *Y. lipolytica* W 29 from the collection of CIRM Levures (France) was used in this study. The culture was raised in batches of 100 mL in glycerol (1%)-containing a medium of the following composition (g/L): MgSO_4_—0.5, (NH_4_)_2_SO_4_—0.3, KH_2_PO_4_—2.0, K_2_HPO_4_—0.5, NaCl—0.1, CaCl_2_—0.05. Then, 2M KP_i_ stock buffer was prepared by dissolving KH_2_PO_4_ anhydrous (272 g/L, Amresco Cat # 0781), pH adjusted with 2M K_2_HPO_4_ to 6.0. Further, 2 M KP_i_ stock buffer was prepared by dissolving anhydrous K_2_HPO_4_ (342 g/L, Amresco Cat # 0705), pH adjusted with 2 M KH_2_PO_4_ to 9.0 Both KP_i_ buffers were sterilized by autoclaving and added to sterilize the culture medium (ratio 1:40) just before inoculation. The yeast was cultivated at different ambient pH values at temperatures of 29 °C and 38 °C as described in [20]. The pH of the broth containing the buffer was monitored using a pH meter each time. A pH value of 5.5 was the actual pH of the medium after adding the buffer. The yeast biomass was collected at the stage of the stationary growth phase (24 h of cultivation), which corresponded to the optical density (OD) of the cell suspension at a wavelength of 595 nm, 9.0–10.0 units. Absorbance (A) was assessed in cell suspensions at a wavelength of 590 nm (A_590_) using a Specol-11 spectrophotometer (Germany). 

### 5.2. Detection of ROS

The dynamics of intracellular total ROS production was monitored using a spectroscopic fluorescence probe of dihydro-2,7-dichlorofluorescein diacetate ester (H_2_DCF-DA) (Sigma, Saint-Luis, MO, USA) as described previously [65].

### 5.3. Cell Respiration

Oxygen consumption by yeast cells was assessed in vitro at 25 °C using oxygen Clarke electrodes coated with a fluoroplastic film at a constant potential of −660 mV. The incubation medium for the experiment contained 50 mM KPi; pH 5.5 and 1% glucose [66].

### 5.4. Preparation of Cellular Homogenate

The cellular homogenate was obtained as follows: cells were washed twice with ice-cold water, and resuspended in grinding medium (1:1 *w*/*v*). The medium contained: 10 mM MES, 0.5 M mannitol, 5 mM EDTA, and 0.5 mM phenyl-methylsulfonyl-fluoride (PMSF); pH 6.5. The yeast cells were disrupted with an ultrasonic disintegrator 9MSE (Farmacia, Stockholm, Sweden) using some pulses at 0 °C for 2 min interrupted by cooling periods every 30 s. The obtained homogenate was centrifuged at 10,000× *g* for 30 min and the supernatant was collected and used for activity determination and further purification [66].

### 5.5. Purification of AH from Y. lipolytica Cells

The purification of the enzyme was carried out in several stages. During the isolation of AH after homogenization of the material from low-molecular-weight compounds, the enzyme was separated by gel filtration on Sephadex G-25. Next, we used column ion-exchange chromatography on DEAE-cellulose. All operations were carried out in a cold chamber at 0–4 °C. 

#### 5.5.1. Gel Filtration on Sephadex G-25

The protein mixture was purified from low-molecular-weight impurities by means of gel filtration through a column with Sephadex G-25 (1.5 × 20 cm) (Fine) from Pharmacia (Sweden). In this process, 0.01 M Tris-HCl buffer (pH 7.8) containing 0.1 mM EDTA, 1% β-mercaptoethanol, and 10 mM Fe^2+^ was used as an elution medium. The elution rate was 20–25 mL/h, and its regulation was carried out by changing the hydrostatic pressure. Each fraction with a volume of 2–3 mL was analyzed for the presence of enzymatic activity. Fractions with the highest enzymatic activity were pooled and used for further purification.

#### 5.5.2. Ion Exchange Chromatography on DEAE-Cellulose

The enzyme was purified by means of ion-exchange chromatography on DEAE-cellulose from Whatman (Great Britain). Before use, ion exchangers were subjected to special treatment. For each gram of dry matter, 30 mL of distilled water was added and left to swell for 3–4 h. Then, the preparation was kept for an hour in 0.5 N NaOH, then in 0.5 N HCl, and again in 0.5 N NaOH. After each treatment stage, the ion exchanger was washed with distilled water until the wash water was neutral. The ion exchanger was placed in a column with dimensions of 1.4 × 10 cm and equilibrated for 8–10 h with an eluting medium. An enzyme preparation previously freed from low-molecular-weight impurities was applied to the columns. The elution medium used in the ion exchange chromatography had the same composition as the buffer used during pre-gel filtration through Sephadex G-25. To purify AH, a stepwise concentration gradient of KCl (100–200 mM) in elution buffer was used.

### 5.6. Measurement of AH Activity

AH activity was measured in a medium containing 50 mM Tris-HCl buffer (pH 7.8), 4 mM citrate. An increase in extinction at 235 nm was recorded due to the formation of an intermediate reaction product, cis-aconitate, which has a double bond. To determine the activity, 2 mL of the medium and 0.4 mL of the cell homogenate were added to the cuvette. A medium containing no homogenate served as a control. The extinction was measured within 3 min after the addition of the homogenate. Activity was expressed in enzymatic units or as specific activity. A unit of enzymatic activity (E) was taken as the amount of enzyme catalyzing the formation of 1 μmol of the reaction product per 1 min at a temperature of 25 °C. 

AH activity was calculated using the following formula:

E = D × 2.0 × V/∆V × τ × 3.09

where D is the increase in optical density at 235 nm over a certain time; 2.0 is the volume of the solution in the cuvette, mL; V is the total volume of the enzyme solution, mL; ∆V is the volume of the sample introduced for measurement, mL; τ is the measurement time, min; and 3.09 is the extinction coefficient corresponding to the absorption value, which is given by 1 μmol of cis-aconitate, located in 1 mL of the test mixture when measured on a spectrophotometer, when the layer thickness of the measured solution is 1 cm.

### 5.7. Determining the Amount of Protein

Total protein was determined by the biuret method. The optical density of solutions was determined on a spectrophotometer at 550 nm. 

### 5.8. Statistical Processing of Experimental Data

The experiments were carried out in 5–6 independent biological replicates, and analytical replicates for each sample were performed in duplicates. The results of the experiments were mean values and their standard deviations compared with the corresponding controls. To calculate the statistical significance of the differences in the results, the method of variation statistics was used. The data obtained were processed using paired *t*-tests. Statistically significant differences at *p* < 0.05 were discussed. Statistical data processing was carried out on IBMPC/AT using Stadia software (version 8.0).

## Figures and Tables

**Table 1:** The results of the purification of AH from *Y. lipolytica* cells of the studied groups *.

Stage of Purification	Group	Total Activity, Units	The Protein Amount, mg	Specific Activity, E/mg Protein	Yield, %	Degree of Purification
Homogenate	1 (pH 4.0)	2.25 ± 0.101	250 ± 11.5	0.009 ± 0.0003	100	1
	2 (pH 5.5)	2.79 ± 0.112 ^a,b^	233 ± 10.8 ^a^	0.012 ± 0.0003 ^a^	100	1
	3 (pH 9.0)	1.94 ± 0.096 ^b^	277 ± 12.3 ^b^	0.007 ± 0.0002 ^b^	100	1
Chromatography on sephadex G-25	1 (pH 4.0)	1.77 ± 0.082	183 ± 9.2	0.015 ± 0.0006	79	1.7
	2 (pH 5.5)	2.29 ± 0.111 ^a,c^	111 ± 5.2 ^a^	0.018 ± 0.0007 ^a^	82	1.5
	3 (pH 9.0)	1.43 ± 0.061	182 ± 8.5	0.012 ± 0.0006	74	1.7
Chromatography on DEAE cellulose	1 (pH 4.0)	0.765 ± 0.034 ^c^	1.77 ± 0.08 ^c^	0.432 ± 0.0212 ^c^	34	48
	2 (pH 5.5)	0.865 ± 0.042 ^d^	1.57 ± 0.07	0.552 ± 0.0271	31	46
	3 (pH 9.0)	0.357 ± 0.015 ^d^	1.58 ± 0.07	0.357 ± 0.0158 ^d^	29	51

* Note: Table discusses statistically significant differences at: ^a^—*p* < 0.01; ^b^—*p* < 0.0004; ^c^—*p* < 0.03; ^d^—*p* < 0.003.

**Table 2:** Kinetic parameters of the catalytic action of AH from *Y. lipolytica* when grown under conditions of different pH values *.

Growth Conditions	Citrate		Isocitrate		pH-Optimum
	V_max_, mmols/mg of Protein-	K_m_, mM	V_max_, mmols/mg of Protein-	K_m_, mM	
pH 4.0	0.570 ± 0.042 ^a^	0.230 ± 0.009 ^b,c^	0.415 ± 0.017 ^a^	0.150 ± 0.008 ^c^	7.6
pH 5.5	0.720 ± 0.031 ^c^	0.320 ± 0.011	0.726 ± 0.037 ^c^	0.270 ± 0.015 ^b,c^	8.0
pH 9.0	0.428 ± 0.022 ^a^	0.190 ± 0.007 ^b^	0.394 ± 0.016 ^a^	0.140 ± 0.006 ^c^	8.2

* Note: The table discusses statistically significant differences at *p* ≤ 0.05. ^a^—*p* < 0.004; ^b^—*p* < 0.003; ^c^—*p* < 0.001. Values are mean ± standard deviation from 5–6 independent experiments.

## Data Availability

Additional data may be available upon personal request.
